# Antibacterial Evaluation of Lithium-Loaded Nanofibrous Poly(L-Lactic Acid) Membranes Fabricated via an Electrospinning Strategy

**DOI:** 10.3389/fbioe.2021.676874

**Published:** 2021-04-29

**Authors:** Chaoan Liang, Qiming Jiang, Yi Yu, Tao Xu, Hanyu Sun, Feilong Deng, Xiaolin Yu

**Affiliations:** ^1^Guangdong Provincial Key Laboratory of Stomatology, Department of Oral Implantology, Hospital of Stomatology, Guanghua School of Stomatology, Sun Yat-sen University, Guangzhou, China; ^2^Biomanufacturing and Rapid Forming Technology Key Laboratory of Beijing, Key Laboratory for Advanced Materials Processing Technology, Ministry of Education, Department of Mechanical Engineering, Tsinghua University, Beijing, China

**Keywords:** anti-bacterial, barrier membranes, lithium, electrospinning, nanofibers

## Abstract

Lithium (Li) reportedly has anti-bacterial properties. Thus, it is an ideal option to modify barrier membranes used for guided bone regeneration to inhibit the bacterial adhesion. The aims of this study were to fabricate and characterize nanofibrous poly(L-lactic acid) (PLLA) membranes containing Li, and investigate their antibacterial effects on *Porphyromonas gingivalis* and *Actinobacillus actinomycetemcomitans in vitro*. Li (5%Li, 10%Li, and 15%Li)-loaded nanofibrous PLLA membranes were fabricated using an electrospinning technique, and characterized via scanning electron microscopy, X-ray photoelectron spectroscopy, X-ray diffraction, a contact angle measuring device, and a universal testing machine. Sustained release of Li ions was measured over a 14-day period and biocompatibility of the Li-PLLA membranes was investigated. Evaluation of bacterial adhesion and antibacterial activity were conducted by bacterial colony counting, LIVE/DEAD staining and inhibition zone method using *P.gingivalis* and *A.actinomycetemcomitans*. Of the three Li-loaded membranes assessed, the 10%Li-PLLA membrane had the best mechanical properties and biocompatibility. Adhesion of both *P.gingivalis* and *A.actinomycetemcomitans* on Li-PLLA membranes was significantly lower than adhesion on pure PLLA membranes, particularly with regard to the 10%Li and 15%Li membranes. Significant antibacterial activity of Li-PLLA were also observed against according to the inhibition zone test. Given their better mechanical properties, biocompatibility, and antibacterial activity, PLLAs with 10%Li are a better choice for future clinical utilization. The pronounced antibacterial effects of Li-loaded PLLA membranes sets the stage for further application in guided bone regeneration.

## Introduction

Dental implant therapy is a good choice for patients with partial or complete edentulism. Patients benefit from evolving clinical concepts and strategies by way of better treatment and improved quality of life. One such strategy is guided bone regeneration (GBR) for localized alveolar defects, a technique that uses barrier membranes to prevent epithelial cells and fibroblasts from growing into defects, while ensuring the growth of osteoblasts and blood vessels (Nyman, 1991; Hammerle and Jung, 2003). Numerous animal experiments (Dahlin et al., 1989; Lee et al., 2020) and human clinical studies (Buser et al., 1990; Wachtel et al., 1991; Lang et al., 1994) indicate that GBR is a successful method for augmenting bone in defected areas. Notably, however, postoperative complications can occur due to the complexity of the oral environment. In a recent meta-analysis the incidence of soft tissue complications after GBR procedures was 16.8% (Lim et al., 2018), including membrane exposure, soft tissue dehiscence, and acute infection/abscess. *Porphyromonas gingivalis* (*P.g*) and *Actinobacillus actinomycetemcomitans* (*A.a*) are reportedly the main pathogens that contribute to such infections and inflammation (Kornman and Robertson, 2000) which hinder the process of bone regeneration (Simion et al., 1994). Thus, GBR membranes with antibacterial properties are needed to promote better prognoses.

Lithium (Li) is a well-known treatment for bipolar and other psychiatric disorders (Oruch et al., 2014). It is a microelement needed in minute quantities to maintain bodily functions including metabolism, neural communication, and cell proliferation (Schrauzer, 2002; Shahzad et al., 2017) but few studies have investigated the antibacterial and immunopotentiating properties of Li (Shenkman et al., 1980; Zhang et al., 2009; Kalinowska et al., 2011; Tay et al., 2012; Moghanian et al., 2018). In the recent study, Li was substituted for calcium in bioactive glass and reported antibacterial effects *in vitro* (Moghanian et al., 2018). Antibacterial effects of Li in mice infected with *Francisella tularensis* (Zhang et al., 2009) and *Burkholderia pseudomallei* (Tay et al., 2012) were also, respectively, reported. Thus Li may be an antibacterial substance, but its effects on *P.g* and *A.a* have not been reported.

Recent studies have shown that Li can induce bone formation and osseointegration via the canonical Wnt/β-catenin signaling pathway (Huang et al., 2019). It has also been reported that Li can inhibit the process of osteoclastogenesis (Arioka et al., 2014) and the formation of multinuclear cells in long-term human marrow cultures (Pepersack et al., 1994). Li is therefore a therapeutic option for bone regeneration due to multiple properties.

To obtain GBR membranes for bone regeneration with desirable properties such as biocompatibility, biodegradability, osteoconductivity, osteoinductivity, and antibacterial capacity, electrospinning and related techniques utilizing synthetic and/or natural polymers have attracted increasing interest (Bottino et al., 2012; Shahriar et al., 2019). Electrospinning is a relatively simple and unique method for fabricating nanofibrous membranes (Rodriguez-Tobias et al., 2019). The nanofibers used have a high surface area-to-volume ratio, a controllable degradation rate, favorable mechanical properties, and high porosity. Various materials have been used to generate electrospun fibrous membranes, including poly(L-lactic acid) (PLLA) which has been approved by the Food and Drug Administration for human clinical use due to its outstanding biocompatibility and biodegradability. Wang et al. (2017) developed conductive nanofibrous sheets based on polylactide and polyaniline via electrospinning and indicated their great potential in cardiac tissue engineering and bioactuators applications. Shi et al. (2016) also developed biodegradable PLLA patches with a 3D network structure and reported positive results when they were used for dural repair.

The electrospinning technique was deemed suitable for fabricating the antibacterial membranes used in the current study, and PLLA eventually degrades and creates an acidic environment that may inhibit bacterial growth (Zilberman and Elsner, 2008). In the present study, Li-loaded nanofibrous PLLA membranes were fabricated via electrospinning techniques. The aim of the study was to characterize these membranes and investigate their antibacterial effects on *P.g* and *A.a in vitro*.

## Materials and Methods

### Composite Material and Membrane Preparation

Reagents were obtained from Aladdin (China) unless otherwise stated. PLLA (PURAC Biochem, Netherlands) was first dissolved in a mixture of hexafluoroisopropanol and N,N-dimethylformamide (4/1, v/v) at a concentration of 6% w/v, into which Li chloride was added. Li chloride concentrations of 5, 10, and 15% tested. The mixtures were stirred at room temperature. A total of four groups were included in the study; PLLA-only (PLLA), 5% LiCl (5%Li), 10% LiCl (10%Li), and 15% LiCl (15%Li).

Each polymer solution was loaded into a 10-mL syringe. The electrospinning setup consisted of a high-voltage power supply and a digitally controlled syringe pump. The electrospun nanofibrous membranes were collected on an aluminum foil roller. During the process of electrospinning, the distance between the tip of the syringe and the collector was set at 20 cm, and the rotation speed of the roller was set at 100 rpm. The flow rate of the solution was set at 0.2 mL/h, and the applied voltages were set at a range of 15–20 kV. The fibers were deposited layer by layer as a fleece-like structure. The membranes were cut into disks with a diameter of 1 cm.

### Characterization of the Nanofibrous PLLA Membranes

#### Membrane Morphology

The electrospun nanofibrous PLLA and Li-PLLA membranes were coated with gold and their morphologies were assessed via field-emission scanning electron microscopy (SEM) (S-4800, Hitachi, Tokyo, Japan) at 2 kV. Image-Pro Plus (Rockville, MD, United States) was used to measure fiber diameters and distributions quantitatively.

#### Elemental Compositions and Phase Analysis of Membranes

Chemical elements were assessed via X-ray photoelectron spectroscopy (XPS) (Thermo Scientific ESCALAB 250Xi, United States). X-ray diffraction (XRD) (X’Pert-PRO, PANalytical, Netherlands) patterns were obtained over a 2θ range of 5°–70° at 40 kV and 40 mA (Cu Kα).

#### Tensile Strength

The effects of Li incorporation on mechanical membrane performance were tested by evaluating tensile strength with a universal testing machine (Instron 5967, United States). Samples of membrane with a thickness of 0.1 mm were cut into 5 × 20-mm rectangular strips and tested under a constant upper clamp speed of 10 mm/min. Three samples were tested in each group and their tensile strengths were analyzed. The elastic modulus was calculated from the slope of the linear region (ε = 1–4%) of the tensile-stress curve.

#### Contact Angles

The contact angles of the nanofibrous membranes were measured via the sessile-drop method with an optical contact angle measuring device (OCA40 Micro, Dataphysics, Stuttgart, Germany).

#### Ion Release From Nanofibrous Li-PLLA Membranes

Li ion release from the samples was determined by assessing changes in ion concentration in pH 7.4 phosphate-buffered saline (PBS) at various timepoints. Samples of 5%Li, 10%Li, and 15%Li were added to PBS at 37°C for 14 days. The PBS was changed every day, and the cumulative ion concentrations at each timepoint were assessed via inductively coupled plasma optical emission spectroscopy (ICP-OES) (Optima 8000DV, Perkin Elmer, United States).

#### Cell Viability Assay

MC3T3-E1 cells were seeded onto samples in 48-well plates at a density of 1.5 × 10^4^ cells**⋅**mL^–1^ and cultured in α-MEM for 1, 4, and 7 days. At each timepoint the Cell Counting Kit-8 assay (CCK-8) (Dojindo, Kumamoto, Japan) was used to assess cell proliferation. One hundred microliters of 10% CCK-8 fluid with medium was added to each well, then the cells were incubated for 2 h. The optical absorbance of the CCK-8 fluid was then measured at 450 nm.

### *In vitro* Bacterial Adhesion and Antibacterial Activity

#### Bacterial Culture and Adhesion

*P.g* (ATCC 33277) and *A.a* (ATCC 43717) were used in bacterial adhesion assays. *P.g* was inoculated on brain heart infusion (BHI) agar plates containing sheep blood and vitamin K1. *A.a* was inoculated on BHI agar plates. After incubation under anaerobic conditions at 37°C for 72 h, single colonies of *P.g* and *A.a* were, respectively, transferred to 15-mL centrifuge tubes containing 10 mL medium. After incubation under anaerobic conditions at 37°C for another 72 h, the bacterial suspensions were diluted to approximately 1 × 10^7^ colony forming units (CFU)/mL for bacterial adhesion assays and antibacterial activity assessment using the McFarland Scale.

All samples were placed in 48-well non-tissue culture plates and 1 mL of diluted bacterial suspension was added to each sample. After incubation under anaerobic conditions at 37°C for 72 h the samples were rinsed with PBS twice to remove unbound bacteria, and bacterial colony counting and LIVE/DEAD staining assays were conducted using these samples.

To visualize bacterial adherence in the samples, after a 72-h incubation one sample from each group was immersed in 2.5% glutaraldehyde for 10 h at 4°C then subjected to gradient dehydration using 50, 75, 90, 95, and 100% ethanol. After spraying with gold, the samples were observed via SEM.

#### Bacterial Colony Counting

After incubation, the samples were rinsed with PBS then transferred into Eppendorf tubes containing 3 mL PBS. The tubes were sonicated for 5 min, followed by a 15-s vortex to detach adhered bacteria. The treated solutions were then diluted to suitable concentrations and 100 μL was inoculated onto BHI agar plates. After incubation under anaerobic conditions at 37°C for 72 h, colonies of *P.g* and *A.a* were counted to evaluate the amounts of live adhered bacteria in the samples.

#### Bacterial LIVE/DEAD Staining

The LIVE/DEAD^®^ BacLight^TM^ Bacterial Viability Kit (L7012, Thermo Scientific, United States) was used to distinguish live and dead bacteria. After incubation the samples were rinsed with PBS then incubated with 200 μL LIVE/DEAD^®^ BacLight^TM^ solution prepared in accordance with the manufacturer’s instructions at room temperature in the dark for 15 min. The SYTO 9 in the solution causes the live bacteria to emit green fluorescence, and the propidium iodide in the solution causes dead bacteria to emit red fluorescence. Confocal laser scanning microscopy (CLSM) (Nikon A1 plus, Japan) was used to assess the samples. Image-Pro Plus was used to perform quantitative analysis to evaluate bacterial adhesion based on the determination of fluorescence intensity in each captured image. Results were obtained from three captured images and normalized to the green/total fluorescence intensity of PLLA samples and red fluorescence intensity of 15%Li samples.

#### Assessment of Antibacterial Activity

The inhibition zone method was used for gaging the antibacterial activity of samples (Zhang et al., 2019). One hundred microliters diluted bacterial suspension (1 × 10^7^ CFU/mL) of was uniformly spread across the BHI agar plates, followed by placing sterilized samples onto them. After incubation under anaerobic conditions at 37°C for 72 h, the presence of inhibition zones were recorded and the diameters of them were measured. The experiment was repeated three times.

### Statistical Analysis

Statistical analysis was performed using SPSS software, version 23.0 (IBM SPSS, United States). The level of significance was determined by one-way analysis of variance followed by Fisher’s least significant difference test for multiple comparisons. The significance level was set at *p* < 0.05.

## Results

### Membrane Characterization

#### Membrane Morphology

**FIGURE 1:**
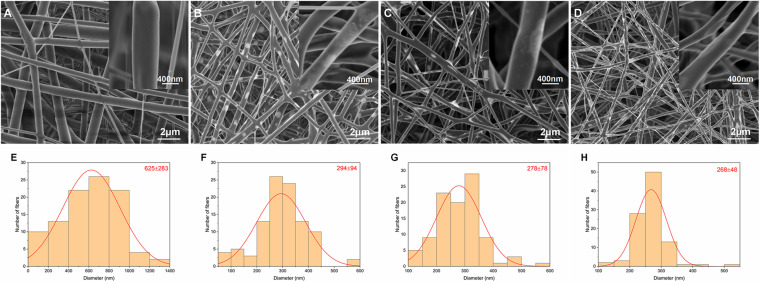
SEM images and corresponding diameter distributions of nanofibrous PLLA **(A,E)**, 5%Li **(B,F)**, 10%Li **(C,G)**, and 15%Li **(D,H)** membranes.

The morphologies and corresponding fiber diameter distributions in the four groups are shown in Figure 1. Fibers exhibited a randomly oriented morphology and 3D network structure. Li was visible as white patches in the fibers. The mean fiber diameter tended to decrease with increasing incorporation of Li. This was due to reduced solution viscosity and increased solution conductivity.

#### Elemental Composition and Phase Analysis

**FIGURE 2:**
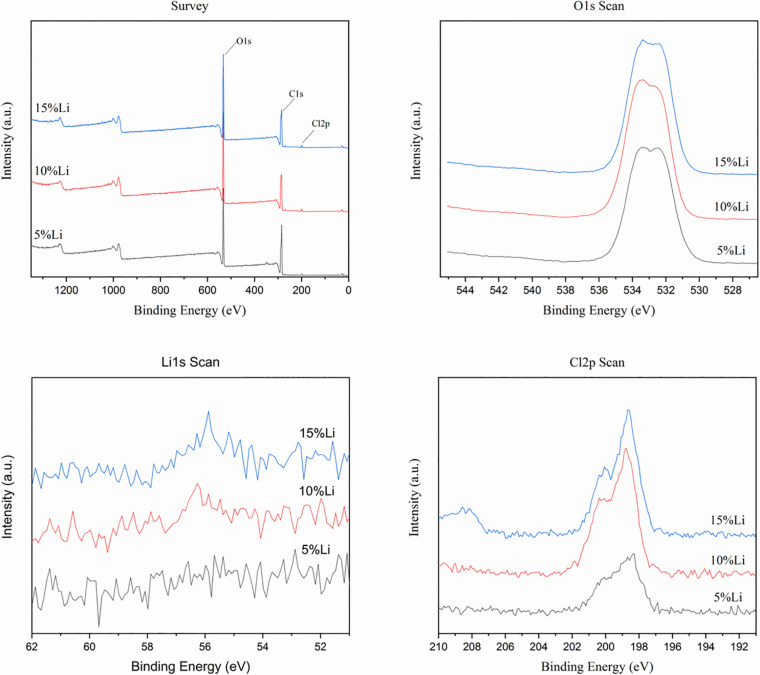
XPS spectrums of 5%Li, 10%Li, and 15%Li membranes.

**FIGURE 3:**
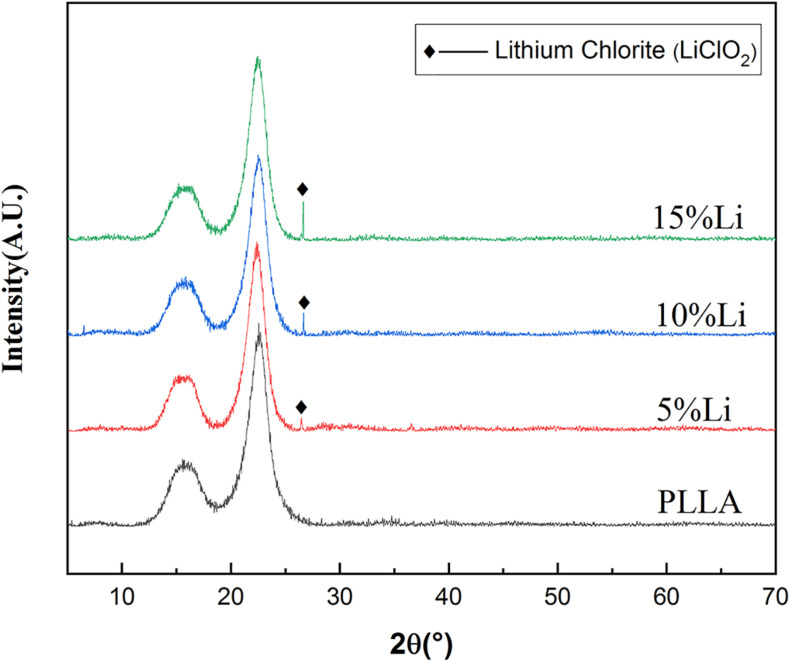
XRD spectrums of PLLA, 5%Li, 10%Li, and 15%Li membranes.

The chemical element compositions of 5%Li, 10%Li, and 15%Li membranes were analyzed via XPS, and the results are shown in Figure 2. All three Li-loaded membranes yielded two peaks at approximately 200.1 and 198.6 eV in the Cl 2p spectra, indicating the presence of Cl^3+^ and Cl^+^, and a peak at approximately 533.5 eV in the O 1s spectra indicating the presence of O^2–^. Peaks at approximately 56.0 eV in the Li 1s spectra of 10%Li and 15%Li membranes indicated the presence of Li^+^. No Li peak was evident in the Li 1s spectrum of the 5%Li membrane, possibly because the Li content was too low. The diffraction patterns of PLLA membranes with different Li content are shown in Figure 3. The XRD spectra of 5%Li, 10%Li, and 15%Li samples exhibited approximate diffraction peaks at approximately 26.5°, corresponding with the lithium chlorite (LiClO_2_) phase, which was concordant with the XPS results. Other diffraction peaks reflected the presence of PLLA polymer.

#### Mechanical Properties of the Membranes

**FIGURE 4:**
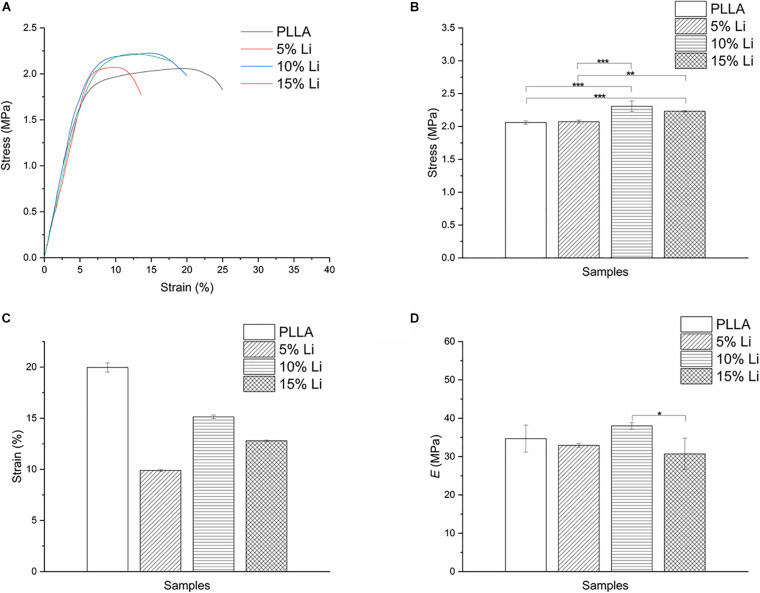
Stress-strain curves **(A)**, tensile strengths **(B)**, strain rates **(C)**, and elastic modulus **(D)** of PLLA, 5%Li, 10%Li, and 15%Li membranes.

Stress-strain curves, maximum tensile strengths, strain rates, and elastic modulus are shown in Figures 4A–D. The PLLA and 5%Li membranes exhibited lower tensile strength than the 10%Li and 15%Li membranes (*p* < 0.01), and the PLLA membrane exhibited a superior strain rate. This revealed that the incorporation of Li improved the mechanical strength of the membranes. The 10%Li membrane had a higher elastic modulus than the 15%Li membrane, and a higher strain rate than the 5%Li and 15%Li membranes.

#### Contact Angle of the Membranes

**FIGURE 5:**
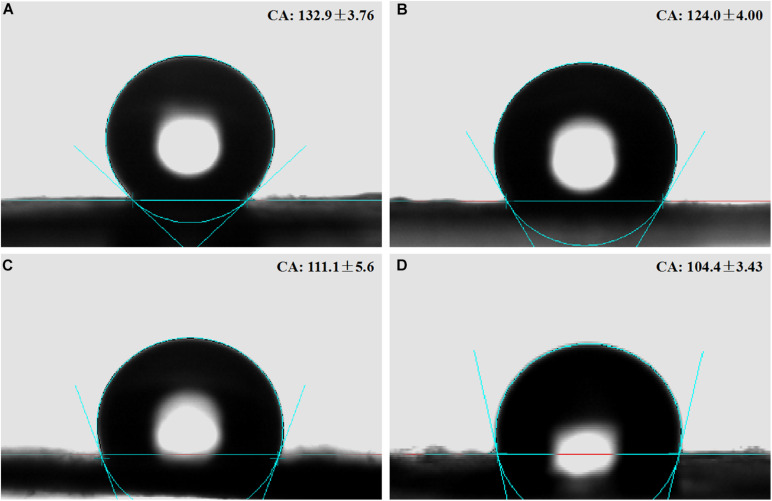
Contact angles on the surfaces of nanofibrous PLLA **(A)**, 5%Li **(B)**, 10%Li **(C)**, and 15%Li **(D)** membranes.

Water contact angles of the membranes were PLLA 132.9° ± 3.76°, 5%Li 124.0° ± 4.00°, 10%Li 111.1° ± 5.62°, and 15%Li 104.4° ± 3.43° (Figure 5 and Table 1). The results indicated that the incorporation of Li improved the hydrophilicity of nanofibrous PLLA membranes (*p* < 0.05).

**TABLE 1:** Water contact angles of the samples.

	Contact angle (°)	*p*-value		
		PLLA	5%Li	10%
PLLA	132.9 ± 3.76	–	–	–
5%Li	124.0 ± 4.00	0.034	–	
10%Li	111.1 ± 5.62	0.000	0.006	–
15%Li	104.4 ± 3.43	0.000	0.001	0.092

#### Ion Release

**FIGURE 6:**
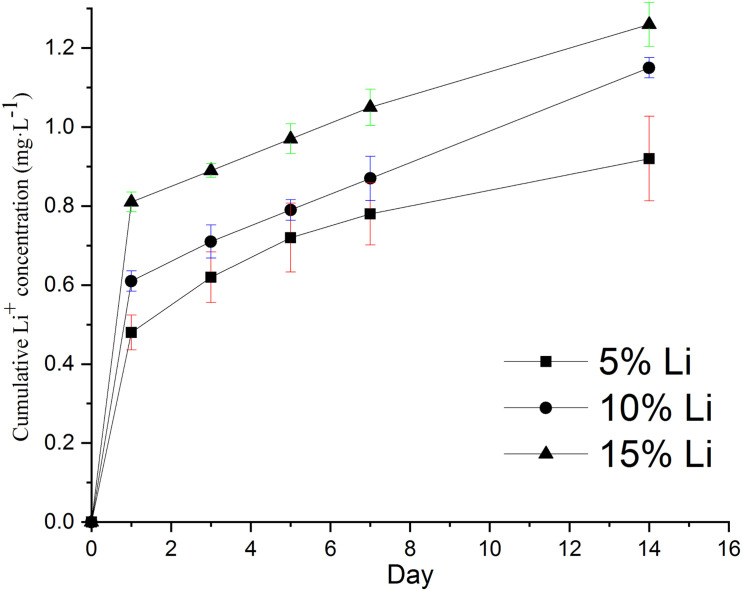
*In vitro* cumulative release concentrations of Li ions from 5%Li, 10%Li, and 15%Li membranes after incubation in PBS at 37°C for 14 d. Data are expressed as means ± the standard deviation (*n* = 3).

Inductively coupled plasma optical emission spectroscopy indicated cumulative release of up to 0.92 mg**⋅**L^–1^ Li^+^ from 5%Li membranes, 1.15 mg**⋅**L^–1^ Li^+^ from 10%Li membranes, and 1.26 mg**⋅**L^–1^ Li^+^ from 15%Li membranes by day 14 (Figure 6). An initial burst of release was evident, followed by sustained release. The release rates of 10%Li and 15%Li membranes were more stable than those of 5%Li membranes, at approximately 0.04 mg**⋅**L^–1^ per day.

#### Cell Viability Evaluation

**FIGURE 7:**
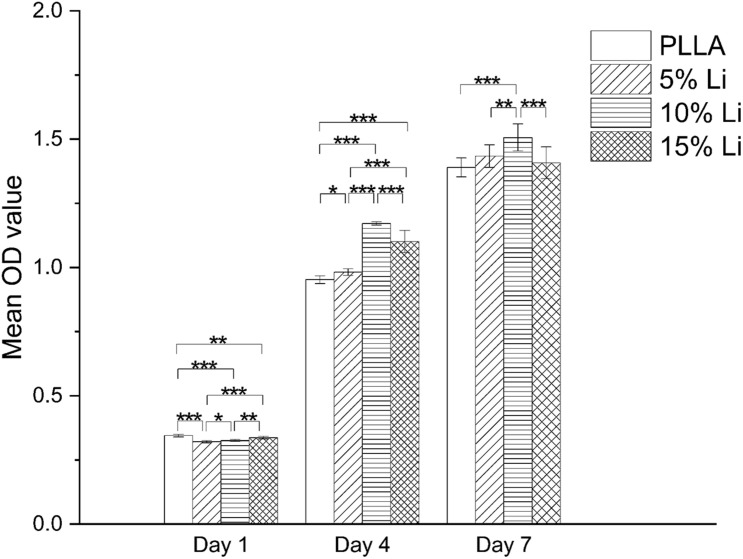
MC3T3-E1 cell proliferation on the surfaces of each membrane after 1, 4, and 7 days. Data are expressed as means ± the standard deviation (*n* = 3). *p < 0.05, **p < 0.01, ***p < 0.001.

The results of the cell viability assay in each group are shown in Figure 7. No toxic effect but pro-proliferative effect was observed at any of the samples. On the 1st day the PLLA and 15%Li groups exhibited more proliferation than the 5%Li and 10%Li groups (*p* < 0.05). On the 4th and 7th days, however, the 10%Li group exhibited more proliferation than any other groups (*p* < 0.001). There were no significant differences between PLLA and 5%Li, PLLA and 15%Li, or 5%Li and 15%Li after 7 days.

### *In vitro* Bacterial Adhesion and Antibacterial Activity

#### Bacterial Adhesion

**FIGURE 8:**
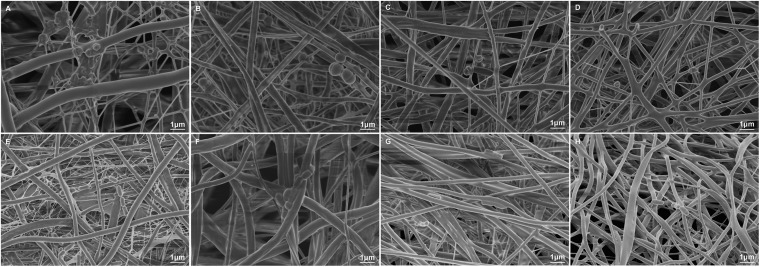
Representative SEM images of *P.g* adhesion on the surfaces of PLLA **(A)**, 5%Li **(B)**, 10%Li **(C)**, and 15%Li **(D)** membranes, and *A.a* adhesion on the surfaces of PLLA **(E)**, 5%Li **(F)**, 10%Li **(G)**, and 15%Li **(H)** membranes after incubation for 72 h.

The results of bacterial adhesion on the membrane surfaces in each group are shown in Figure 8. Representative morphologies of *P.g* and *A.a* were assessed via SEM and there were less bacteria adhering to the 10%Li and 15%Li membranes than to the 5%Li and PLLA membranes. *P.g* tended to form a biofilm on PLLA membranes, whereas a filamentous structure was observed around and between *A.a* on PLLA membranes.

#### Bacterial Colony Counting

**FIGURE 9:**
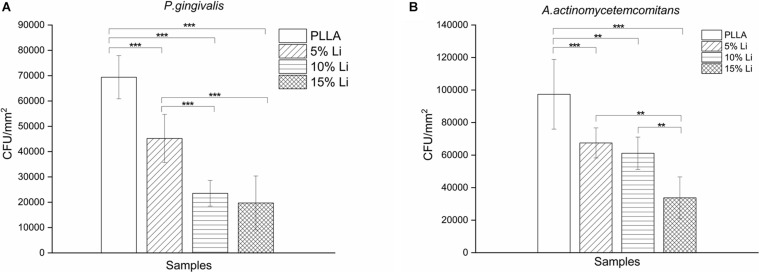
Colony counting of *P.g* **(A)** and *A.a* **(B)** adhering onto the membranes. Data are expressed as means ± the standard deviation (*n* = 6). **p < 0.01, ***p < 0.001.

The results of bacterial colony counting assays are shown in Figure 9. After a 72-h incubation there were significantly more *P.g* and *A.a* CFU on PLLA membranes than on any of the three Li-loaded membranes. There was no statistically significant difference between the *A.a* CFU on 5%Li and 10%Li membranes, or the *P.g* CFU on 10%Li and 15%Li membranes.

#### Bacterial LIVE/DEAD Staining

**FIGURE 10:**
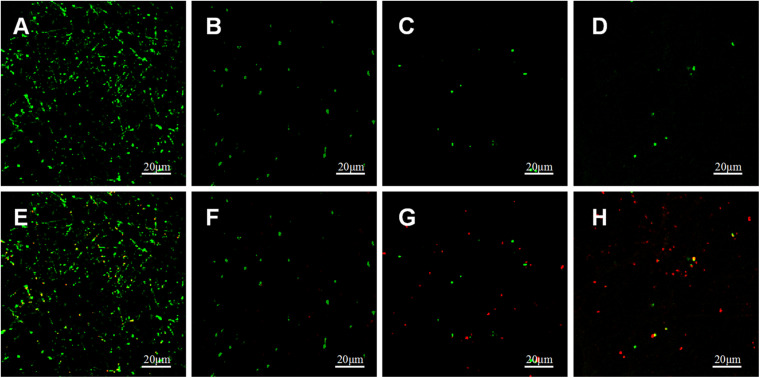
CLSM images of live (green) and dead (red) stained *P.g* adhered on PLLA **(A,E)**, 5%Li **(B,F)**, 10%Li **(C,G)**, and 15%Li **(D,H)** membranes after incubation for 72 h.

**FIGURE 11:**
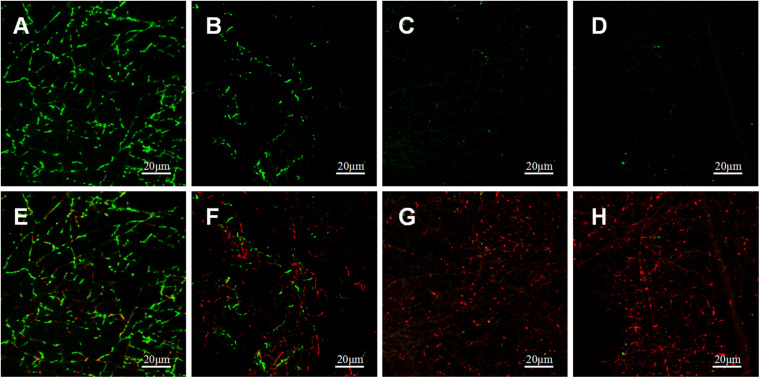
CLSM images of live (green) and dead (red) stained *A.a* adhered on PLLA **(A,E)**, 5%Li **(B,F)**, 10%Li **(C,G)**, and 15%Li **(D,H)** membranes after incubation for 72 h.

**FIGURE 12:**
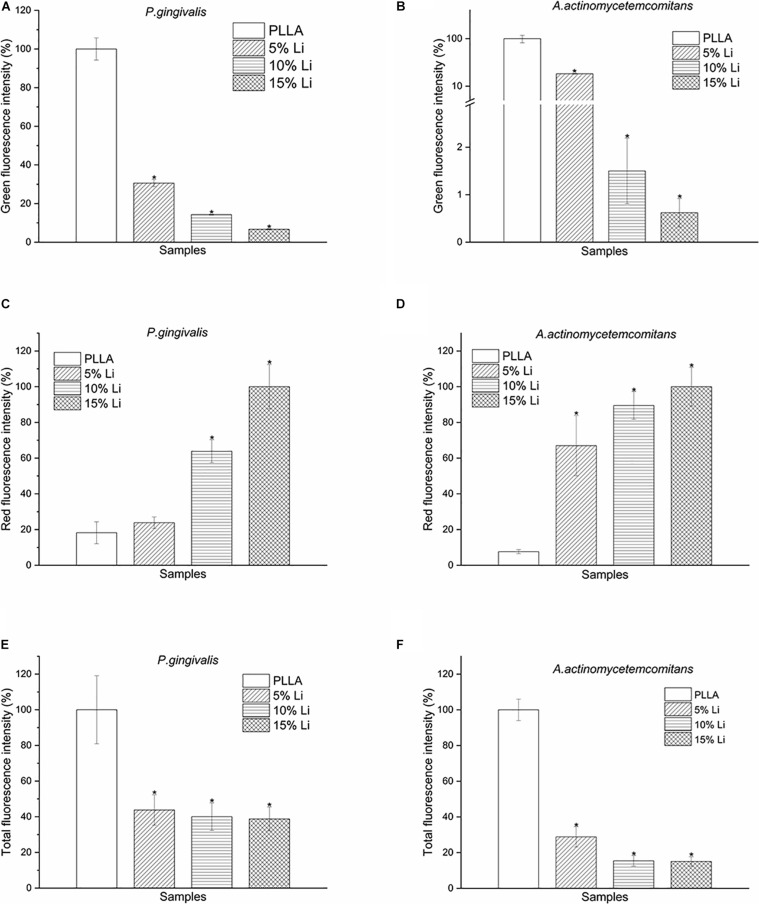
Green **(A,B)**, red **(C,D)**, and total **(E,F)** fluorescence intensity of *P.g* and *A.a* LIVE/DEAD staining after incubation for 72 h. Data are expressed as means ± the standard deviation (*n* = 3). *p < 0.05 compared with bacteria adhered to nanofibrous PLLA membrane.

Adherence of *P.g* and *A.a* after 72 h were assessed via CLSM after LIVE/DEAD staining, and there were differences in the ratios of live/dead bacteria on the surfaces of each sample (Figures 10, 11). Viable *P.g* and *A.a* occupied almost every fiber on the surface of PLLA membranes. Results of quantitative analysis are shown in Figure 12. Increased Li content was associated with significantly lower numbers of viable bacteria and significantly higher numbers of dead bacteria. These results were consistent with the results of the bacterial colony counting assays. Thus, Li-loading had antibacterial effects against *P.g* and *A.a*.

#### Assessment of Antibacterial Activity

**FIGURE 13:**
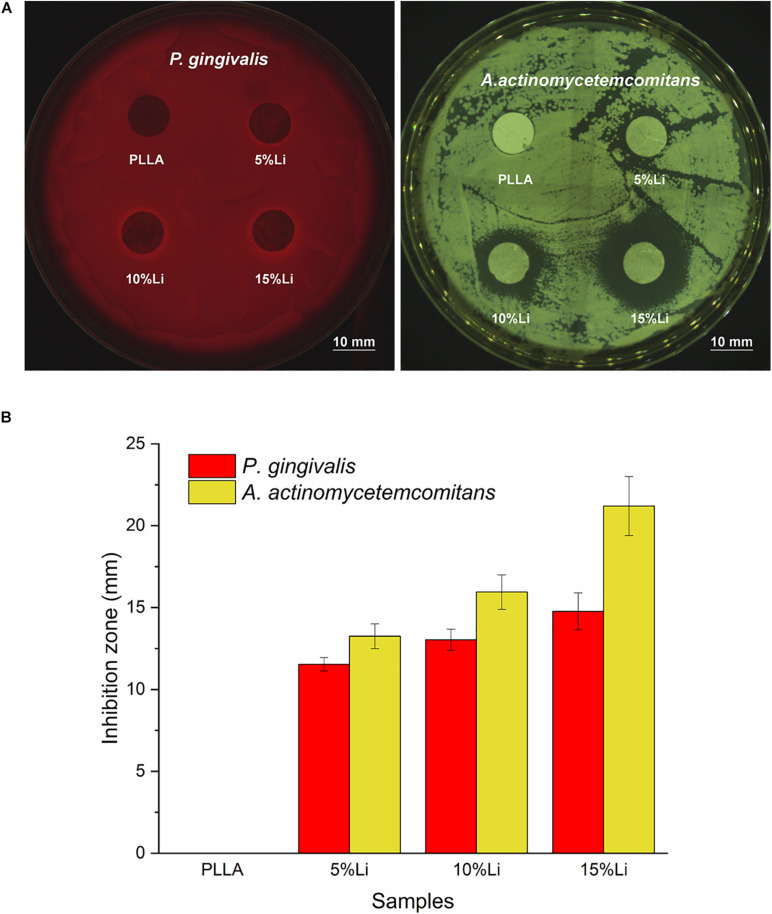
Inhibition zone around the samples **(A)**, mean diameter (mm) of inhibition zone for each sample against *P.g* and *A.a* **(B)**. Data are expressed as means ± the standard deviation (*n* = 3).

The antibacterial activity of the Li-loaded nanofibers was evaluated based on disk diffusion against *P.g* and *A.a*, as shown in Figure 13A. Inhibition zone was observed around 5%Li, 10%Li, and 15%Li, confirming the growth inhibition of *P.g* and *A.a*. The diameters of inhibition zone were measured in millimeters and presented in Figure 13B.

## Discussion

Membranes used in GBR are expected to have multiple functions such as inducing proliferation/differentiation of osteoblasts while inhibiting that of osteoclasts, and antibacterial properties. Several reports indicate that Li that can promote bone formation (Zamani et al., 2009; Chen and Alman, 2009; Cai et al., 2015; Zhang et al., 2018), and a few (Tay et al., 2012; Moghanian et al., 2018) imply that it has antibacterial potential, thus it may be suitable for improving membrane properties. To our knowledge, Li has not been incorporated into electrospun membranes before. In the current study, Li-loaded nanofibrous PLLA membranes with a 3D network structure were generated via an electrospinning technique. Li was successfully electrospun into the fibers, which subsequently exhibited Li ion release. The Li-loaded membranes also exhibited good biocompatibility and antibacterial properties against *P.g* and *A.a*.

Based on the electrospinning technique, Bartnikowski et al. (2018) fabricated a 3D printed polycaprolactone scaffold with Li incorporated into it and reported delayed leaching and immunomodulatory effects of bioactive ions. In the present study, parameters such as solution viscosity, Li concentration, and flow rate were manipulated to generate stable nanofibrous membranes with uniformly distributed Li. Under SEM, Li was visualized as white patches or spots, similar to other metals (Jin et al., 2018; Rajzer et al., 2019) when loaded into fibers. The diameter distribution also suggested that the addition of Li may assist the fabrication of finer fibers with improved mechanical properties (Kidoaki et al., 2006). To prove this, we evaluated the mechanical properties of each group via tensile tests. 10%Li and 15%Li exhibited higher maximum tensile strengths, indicating that the addition of Li enhanced the mechanical strength of the membranes. 10%Li had a higher elastic modulus than 15%Li, suggesting better properties in this regard. We believe that the reason for the higher elastic modulus of 10%Li is related to the stability of facbricating process. An increased Li concentration may result in excessively higher conductivity and lower viscosity of the solution, leading to unstable fabrication and droplet formation on the membranes (Kidoaki et al., 2006; Stachewicz et al., 2012). More Li will be accumulated in the droplets and less will be incorporated into the fibers, resulting in a non-uniform distribution. In the present study, 15%Li resulted in a relatively stable electrospinning process, indicating that that concentration of Li was in the appropriate range. However, comparing to 10%Li, 15%Li probably caused less uniform distribution of Li, resulting in less elastic modulus. In a previous study, fibers with smaller diameters had higher strength but lower ductility (Stachewicz et al., 2012), and similar results were observed in the current study in which the PLLA membrane exhibited a superior strain rate. The 10%Li membrane exhibited greater ductility than the 5%Li and 15%Li membranes, further illustrating the advantages of 10%Li.

The hydrophilicity of the nanofibrous membranes was improved by Li incorporation. The contact angle of the membranes decreased with increasing incorporation of Li. The improved hydrophilicity meets the requirement of barrier membranes, but notably a higher Li content is not necessarily better as mentioned above.

The results of cumulative Li release were as expected. At the early stage there was an initial burst of release, which may assist the avoidance of bacterial contamination during the first 6 h after GBR (Hetrick and Schoenfisch, 2006). Subsequent sustained Li release from the membranes over 14 d has the potential to further prevent bacterial colonization. In the current study the cumulative Li concentration was always within the safe limit (Shahzad et al., 2017; Medic et al., 2020), namely 1.2 mM for humans. This and the results of the CCK-8 assays attest to the biological safety of 3D printed Li-loaded nanofibrous membranes. The results of CCK-8 assays also revealed that the incorporation of Li had a tendency to promote MC3T3-E1 cell proliferation, which is concordant with previous studies (Clement-Lacroix et al., 2005; Liu et al., 2018), implying the medical potential of the membranes. The capacity of the 10%Li membranes to promote cell proliferation was greatest at the 7-day timepoint, again demonstrating the importance of the appropriate Li concentration. Notably, however, the *in vivo* biological safety and pro-proliferative effects of Li-loaded membranes require further study.

Prevention of pathogenic bacterial adhesion can reduce the occurrence of infection and promote a stable prognosis after surgery. One purpose of the current study was to evaluate the antibacterial effects of nanofibrous Li-PLLA membranes. The hydrophilicity of a material’s surface is one of the factors that affects bacterial adhesion. Generally, hydrophilic surfaces have higher surface free energy and attract more bacteria with high surface free energy. Early in 1995, Quirynen and Bollen (1995) investigated the influence of tooth surface on supragingival and subgingival plaque formation, and concluded that increased surface free energy resulted in faster colonization and maturation of plaque. In the present study the improved hydrophilicity and surface free energy of the nanofibrous membranes may have increased the adherence of bacteria. Bacterial colony counting and LIVE/DEAD staining conducted with *P.g* and *A.a* revealed that there was less bacterial adhesion on Li-PLLA membranes than on PLLA membranes, however, confirming the antibacterial activity of the incorporated Li. For *P.g*, 10%Li and 15%Li had similar antibacterial effects and they were superior to those of 5%Li. For *A.a*, 5%Li and 10%Li had similar effects and they were inferior to those of 15%Li. In addition, the antibacterial activity against *P.g* and *A.a* was also verified by the appearance of inhibition zone. The diameter of inhibition zone increased with the increasing concentration of Li. Generally, the size of inhibition zone related to the susceptibility of bacteria to the test agent. Our results suggested that A.a is more susceptible to Li. Taking biocompatibility into consideration, 10%Li is a better choice for future improvement and clinical utilization. Nevertheless, the optimum concentration of Li should be further investigated, particularly between 10 and 15%.

The exact mechanisms of the effects of Li are not yet clear. In CLSM experiments, membranes with greater Li content exhibited less viable bacteria and more dead bacteria on their surfaces than pure PLLA membranes. This suggests that Li has bactericidal effects or inhibits bacteria adhesion. Moghanian et al. (2018) reported similar results with Li-substituted bioactive glass. Studies (Tallon et al., 2007) have shown that Li may affect proteins involved in adhesion, whose physiochemical properties such as hydrophobicity are related to adhesion. Another possibility is that Li cations induce electrotransformation of bacteria and increase the permeability of cell membranes (Papagianni et al., 2007; Ramon and Fonzi, 2009) causing the accumulation of Li + in the cell cytoplasm and inhibition of pyruvate kinase (Niiya et al., 1980; Umeda et al., 1984). Li ions may also influence ion channels or replace other cations, affecting bacterial membranes. The exact mechanism should be further studied *in vivo* because Li is also associated with immunomodulatory effects.

In the present study, the incorporation of Li had antibacterial effects on *P.g* and *A.a*. It has previously been reported that Li rendered bacteria more sensitive to acid (Gallagher and Cutress, 1977). In the current study PLLA was used as the basic material, and it ultimately degrades and creates an acidic environment, promoting the reduction of bacterial aggregation. The use of Li as a treatment for bone regeneration has been extensively studied, but its antibacterial properties in this context have not. Hence, further confirmation of the antibacterial effects of Li-loaded nanofibrous PLLA membranes and their effects on bone regeneration are required to establish its viability as a barrier membrane for GBR.

## Conclusion

In the present study, Li-loaded nanofibrous PLLA membranes were generated via an electrospinning technique. They exhibited a 3D network structure, sustained release of Li ions, improved mechanical strength, and hydrophilicity. The Li-loaded PLLA fibers significantly reduced the adherence and inhibited the growth of *P.g* and *A.a* compared to PLLA fibers alone, particularly the 10%Li and 15%Li membranes. Given their better mechanical properties and biocompatibility, 10%Li is a high-potential multifunctional candidate in the field of GBR.

## Data Availability Statement

The raw data supporting the conclusions of this article will be made available by the authors, without undue reservation.

## Author Contributions

CL and XY: conceptualization. CL, QJ, and TX: methodology. CL and QJ: software. CL, QJ, YY, and HS: validation. CL: formal analysis. CL and QJ: investigation, writing—original draft preparation. XY and FD: resources and writing—review and editing. FD: supervision. XY: project administration. All authors have read and agreed to the published version of the manuscript.

## Conflict of Interest

The authors declare that the research was conducted in the absence of any commercial or financial relationships that could be construed as a potential conflict of interest.
